# Ad-p53 enhances the sensitivity of triple-negative breast cancer MDA-MB-468 cells to the EGFR inhibitor gefitinib

**DOI:** 10.3892/or.2014.3665

**Published:** 2014-12-11

**Authors:** XINZHAO WANG, HONGKUAN SONG, QIAN YU, QI LIU, LEILEI WANG, ZHAOYUN LIU, ZHIYONG YU

**Affiliations:** 1 School of Medicine and Life Sciences, University of Jinan-Shandong Academy of Medical Sciences, Shandong Cancer Hospital, Jinan, Shandong 250117, P.R. China; 2 Juxian Hospital of Traditional Chinese Medicine, Rizhao, Shandong 276500, P.R. China; 3 Department of Biology, Winship Cancer Institute, Emory University, Atlanta, GA 30322, USA; 4 Shandong Cancer Hospital, Jinan, Shandong 250117, P.R. China

**Keywords:** triple-negative breast cancer, MDA-MB-468 cells, p53, EGFR, targeted therapy, PI3K/Akt

## Abstract

Triple-negative breast cancer (TNBC) accounts for 20% of all molecular subtypes of breast cancer. Neither endocrine nor anti-HER2 molecular targeting treatment yield promising results. At present, epidermal growth factor receptor (EGFR) inhibitor, as a single agent, is unable to obtain encouraging results in the treatment of TNBC, even though most of these tumors overexpress EGFR. In the present study, we used recombinant human p53 adenovirus (Ad-p53) and EGFR inhibitor gefitinib to treat the TNBC cell line MDA-MB-468. The combined treatment of gefitinib and Ad-p53 synergistically inhibited the proliferation of MDA-MB-468 cells; it restrained colony formation, enhanced cellular apoptosis and arrested the cell cycle *in vitro*, and decreased tumor burden of xenografts in nude mice. Western blot analysis revealed that Ad-p53 and gefitinib in combination significantly downregulated the phosphorylation of protein kinase B (p-Akt) and upregulated caspase-9 and cleaved caspase-3, while there were minimal effects on the expression of extracellular signal-regulated kinase (ERK) and phosphorylation of ERK (p-ERK). These results suggest that Ad-p53 may block the PI3K/Akt pathway rather than the Raf/MEK/ERK pathway. Importantly, wild-type p53 was able to reverse the drug resistance of MDA-MB-468 cells to gefitinib through inactivation of the phosphatidylinositol 3-kinase (PI3K)/Akt pathway. The apoptotic activity induced by this combined treatment may be regulated by caspase cascade-dependent activation.

## Introduction

Triple-negative breast cancer (TNBC) is the most invasive subtype and accounts for 20% of all molecular subtypes of breast cancer (1). Owing to its lack of estrogen receptor (ER), progesterone receptor (PR) and human epidermal growth factor receptor 2 (HER2/neu), neither endocrine nor anti-HER2 molecular targeting treatment yield promising results, and standard chemotherapy is the backbone of systemic treatment. Over the past decade, preclinical findings have suggested various protein targets and pathways as possible TNBC treatments, such as growth factor receptors, proteins involved in cellular DNA repair capacities and epigenetic regulation (2). Nevertheless, these novel molecular targeting treatments have achieved little clinical progression and there are no effective therapeutic targets to date available against TNBC (1,3).

EGFR overexpression in breast cancer is correlated with large tumor size, more stem-cell like properties and poor prognosis (4). Its overexpression is present in more than 50% of TNBC cases, which is more frequent than in other subtypes (5). Gefitinib, approved for lung cancers, is a tyrosine kinase inhibitor (TKI) that targets the adenosine triphosphate binding site in the cytoplasmic domain of EGFR (6). Unfortunately, gefitinib has shown little efficacy in most clinical studies of breast cancer (7–9). The complex interplay downstream pathways of EGFR with other cellular components lead to their continued activation and insensitivity toward EGFR inhibitors (10). In this regard, many studies have reported that both the Raf/MEK/MAPK and phosphatidylinositol 3-kinase (PI3K)/Akt signaling pathways together promote the resistance of cancer cells to gefitinib (11,12).

p53 is critical to the induction of cell cycle arrest, DNA repair, cellular apoptosis and senescence in response to a wide array of stimuli (13). Its mutation has been related to the poor prognosis and drug resistance of cancer cells (14,15). The p53 mutation is common in TNBC (16,17), and the mutant p53 endows tumor cells with invasive and metastatic abilities (15,18). Huang *et al* (19) reported that p53 regulates the sensitivity to EGFR inhibitors and induces apoptosis by modulating EGFR downstream signaling in lung cancer cells. Recombinant human p53 adenovirus (Ad-p53), a replication incompetent human type 5 adenovirus whose E1 region is replaced by an expression cassette containing the human wild-type p53 cDNA (20), was shown to restore p53 activity in p53-deficient hepatocytes, therefore inducing G_2_/M arrest and apoptosis. However, its effect was not apparent as a single agent treatment in breast cancer. In the present study, Ad-p53 was used in combination with gefitinib to treat a TNBC cell line *in vitro* and *in vivo*. A significant sensitivity toward gefitinib was observed after p53 activity was restored.

## Materials and methods

### Reagents

MDA-MB-468 cells were purchased from the Cell Bank of Shanghai Institute of Cell Biology, Chinese Academy of Sciences. Dulbecco’s modified Eagle’s medium (DMEM) and fetal bovine serum (FBS) were purchased from Gibco (Grand Island, NY, USA). Gefitinib was obtained from Tocris Bioscience Company (Bristol, UK), minimum purity >98%, and dissolved in 100% dimethyl sulfoxide (DMSO; Fisher Scientific, Pittsburgh, PA, USA). A 100-mM stock solution was prepared and stored at −20°C; gefitinib (Iressa^®^) tablets were kindly provided by AstraZeneca (Macclesfield, Cheshire, UK). Ad-p53 (Gendicine, Shenzhen, China), 1×10^12^ virus particles (VP), was stored at −20°C; Annexin V-FITC apoptosis kit was purchased from BD Biosciences Pharmingen (San Diego, CA, USA). p53, caspase-9, Akt, phosphorylation of protein kinase B (p-Akt) (S473), extracellular signal-regulated kinase (ERK) and phosphorylated ERK (p-ERK) (Y204) were purchased from ImmunoWay Biotechnology (Grand Island, NY, USA). EGFR, GAPDH and cleaved caspase-3 were purchased from Cell Signaling Technology (Beverly, MA, USA). 3-(4,5-Dimethylthiazol-2-yl)-2,5-diphenyltetrazolium bromide (MTT) and the BCA protein assay kit were purchased from Beyotime Institute of Biotechnology (Jiangsu, China).

### Cell culture

The human breast cancer cell line MDA-MB-468 was seeded in DMEM supplemented with 10% FBS, 100 U/ml penicillin, 100 mg/ml streptomycin and 2 mM glutamine. Cells were grown in a humidified atmosphere of 5% CO_2_ at 37°C.

### MTT assay

The MDA-MB-468 cell line was plated in 96-well plates in triplicate with 3×10^3^ cells/well. Next, the cells were treated with Ad-p53 at a multiplicity of infection (MOI) of 100 for 24 h, while the vehicle-treated control cells were incubated with 0.5% of DMSO. Next, fixed-ratio concentrations (0, 1.25, 2.5, 5, 10 and 20 μM) of gefitinib were administered. After 48 h, 20 μl of 5 mg/ml MTT solution was added to each well for 4 h. MTT was carefully aspirated and replaced with 200 μl DMSO/well. Absorbance was measured on a Bio-Rad 680 microplate reader (Bio-Rad, Hercules, CA, USA) at 570 nm, and the results were reported relative to a reference wavelength of 630 nm. The inhibitory rate of growth was calculated according to the following equation: Growth inhibition rate = (1 - the mean OD of the samples/the mean OD of the controls) × 100%.

### Clonogenic survival assay

MDA-MB-468 cells were plated in 6-well plates (300 cells/well) and treated with 3 μl of gefitinib and/or an MOI of 100 of Ad-p53 for 48 h. Then, the cells were washed with phosphate-buffered saline (PBS) and replaced with fresh medium. After 14 days, the cells were fixed with 70% ethanol and stained with crystal violet. The number of colonies (>50 cells) was counted under a microscope. Survival was expressed relative to the untreated controls.

### Measurement of apoptotic cells

The cells were treated with either gefitinib (3 μM) or Ad-p53 (MOI of 100) alone or in combination for 48 h, and then washed twice with cold PBS. An indirect immunofluorescence assay was performed using the Annexin V-FITC apoptosis kit according to the manufacturer’s instructions. The samples were assessed by flow cytometry (FACSCalibur; BD Biosciences) using CellQuest software within 1 h.

### Analysis of the cell cycle distribution

MDA-MB-468 cells (1×10^6^) were treated with gefitinib (3 μM) and/or Ad-p53 (MOI of 100) for 48 h and then fixed in 70% of ethanol. After being washed twice with PBS, the cells were stained with propidium iodide (PI) for 30 min. Flow cytometric analysis was performed on the FACSCalibur (BD, Bedford, MA, USA). ModiFIT software (Topsham, ME, USA) was used to analyze the cell cycle distribution.

### Western blot analysis

Cells were treated with gefitinib (3 μM) and/or Ad-p53 (MOI of 100), and then lysed in RIPA buffer (50 mM Tris pH 7.4, 0.15 M NaCl, 1% Triton X-100, 1% sodium deoxycholate, 0.1% SDS) with 1 mM PMSF after 48 h. Supernatants were recovered, and the total protein concentration was detected using the BCA protein assay kit. The proteins were applied to sodium dodecyl polyacrylamide gel electrophoresis, and electrophoretically transferred onto a PVDF membrane (Amersham Biosciences, Buckinghamshire, UK). Appropriate antibodies were applied to determine the membrane. The band density was normalized to GAPDH.

### Effect of gefitinib and Ad-p53 on the growth of MDA-MB-468 xenografts

Sixteen healthy BALB/C female nude mice (4 weeks old) were obtained from the Beijing HFK Bioscience Company. All animals were housed and treated according to the guidelines outlined by the Institutional Animal Care and Use Committee of Shandong Cancer Hospital and maintained under a germ-free controlled environment. The mice were grown until 6 weeks before being subcutaneously injected in the right axillary with MDA-MB-468 cells (1×10^7^). After the tumor xenograft had grown to 1 cm in diameter, the mice were randomized into four treatment groups: i) vehicle; ii) Ad-p53; iii) gefitinib; and iv) the combination. Ad-p53 (1×10^10^ VP) was dissolved in 100 μl physiological saline. All were injected peri-/intratumorally. Gefitinib (100 mg/kg) was administered via oral gavage. The combined treatment was the same as the single agent treatment. Physiological saline was administered to the vehicle-treated group. The greatest longitudinal diameter (a) and the greatest transverse diameter (b) were measured on day 0, 3, 6, 9, 12 and 14. Tumor volume (TV) was calculated by the following formula: TV = 1/2ab^2^. Tumor inhibition rate (TIR) = (average TV of the vehicle-treated group - average TV of the experimental group)/average TV of the vehicle-treated group × 100%.

### Statistical analysis

Statistical analysis was carried out using SPSS 17.0 statistical software. Significant differences between two groups (Table I) were conducted by t-test. The analysis of variance (>2) was analyzed by one-way analysis of variance (ANOVA) to determine statistical significance. Tukey’s multiple comparison was applied to compare two subsequent samples. All statistical tests were two-sided. P<0.05 was considered to indicate a statistically significant result.

## Results

### Ad-p53 enhances the cytotoxic effect of gefitinib on MDA-MB-468 cells

**Figure 1:**
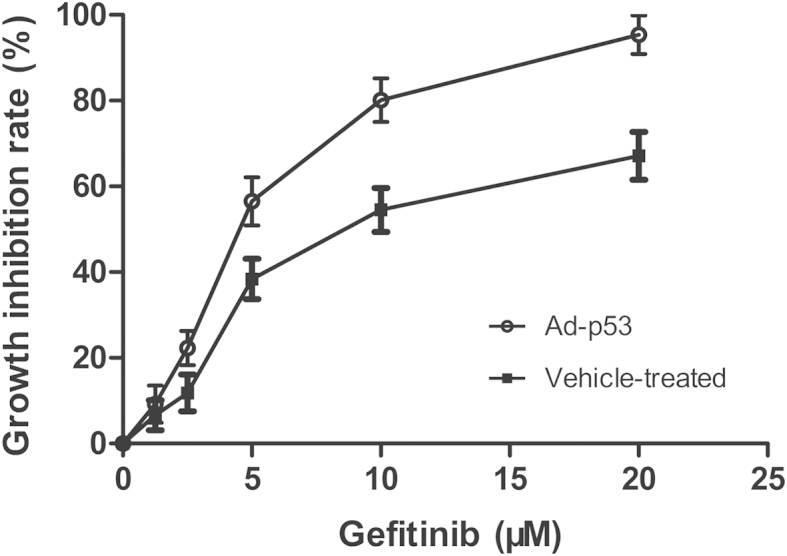
Effects of gefitinib with or without Ad-p53 on the growth of MDA-MB-468 cells. Firstly, cells were infected by Ad-p53 for 24 h; vehicle-treated cells were treated with DMSO. Then, cells with or without Ad-p53 were treated by fixed-ratio concentrations of gefitinib for 48 h, and cell viability was assessed by MTT assay. The results represent means ± SEM from three independent experiments. Ad-p53, recombinant human p53 adenovirus; DMSO, dimethyl sulfoxide.

Gefitinib alone or combined with Ad-p53 inhibited the proliferation of MDA-MB-468 cells in a dose-dependent manner (Fig. 1). Combined treatment of Ad-p53 with gefitinib synergistically inhibited the proliferation of the MDA-MB-468 cells with a lower IC_50_ value of 4.3 μM; in comparison, cells that were not pretreated with Ad-p53 were relatively resistant to gefitinib with a higher IC_50_ value of 8.5 μM (Table I). When extra wild-type p53 gene was integrated into the MDA-MB-468 cells, the cancer cells became more sensitive to gefitinib.

### Combination of Ad-p53 and gefitinib inhibits clonogenic cell survival

**Figure 2:**
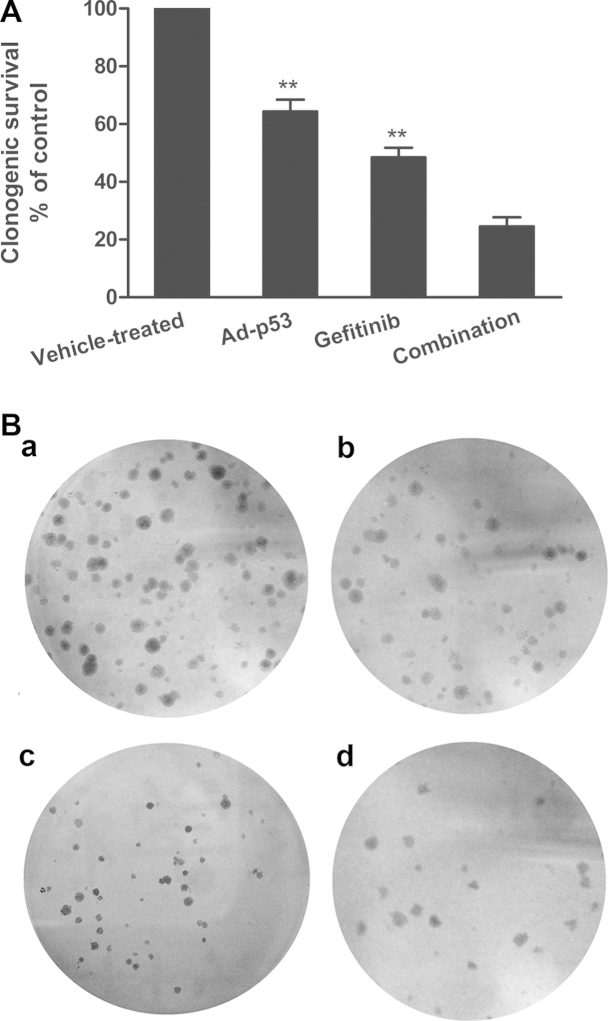
Ad-p53 and gefitinib in combination obviously reduces clonogenic survival. MDA-MB-468 cells were treated with 3 μM of gefitinib alone, MOI of 100 of Ad-p53 alone or in combination for 48 h, and then replaced with new medium. Colonies (>50 cells) were counted after being cultured for 14 days. (A) Survival is expressed relative to the untreated controls. The results represent means ± SEM from three independent experiments. Statistical significance was assessed by ANOVA. Tukey’s multiple comparison was applied to compare two subsequent samples. ^**^P<0.01. (B) Cells stained with crystal violet are presented as follows: a, vehicle treated; b, Ad-p53-treated; c, gefitinib-treated; d, the two drugs combined. Ad-p53, recombinant human p53 adenovirus; MOI, multiplicity of infection.

A clonogenic assay was performed to further investigate the separate and combined effects of Ad-p53 and gefitinib on cell proliferation. Survival was expressed relative to the vehicle-treated cells. Ad-p53 or gefitinib could only slightly weaken colony formation. An approximate 64.4 and 48.5% clonogenic survival rate was detected when cells were treated with Ad-p53 and gefitinib, respectively. Nevertheless, when Ad-p53 and gefitinib were administered in combination, colony formation was significantly decreased with a lower clonogenic survival rate of 24.5% (Fig. 2).

### p53 is required for gefitinib-induced cellular apoptosis and cell cycle arrest

**Figure 3:**
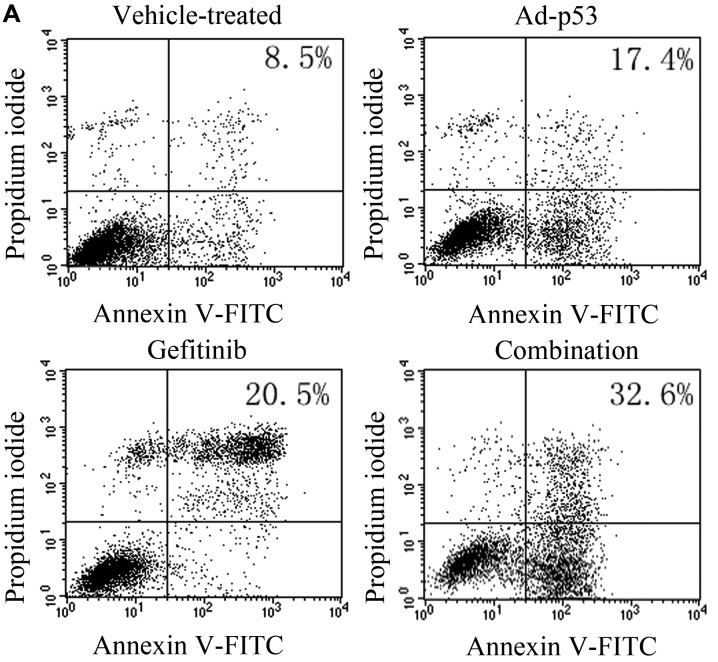

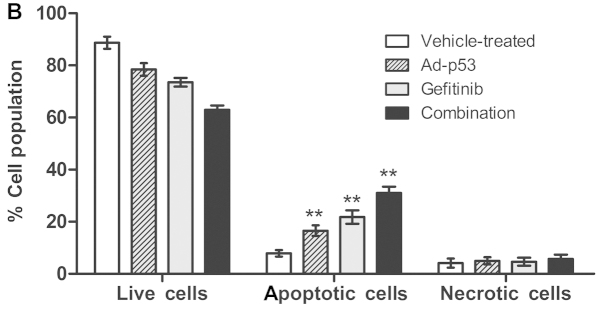
Induction of apoptosis following treatments with 3 μM of gefitinib alone, MOI of 100 of Ad-p53 alone or in combination for 48 h. The apoptosis of MDA-MB-468 cells was detected via Annexin V/FITC using flow cytometry. (A) Percentage of apoptotic cells was obtained from UR and LR panels in each scatter plot for all the treatment groups. (B) Data represent means ± SEM from three independent experiments. Statistical significance was assessed by ANOVA. Tukey’s multiple comparison was applied to compare two subsequent samples. ^**^P<0.01. MOI, multiplicity of infection; Ad-p53, recombinant human p53 adenovirus.

**Figure 4:**
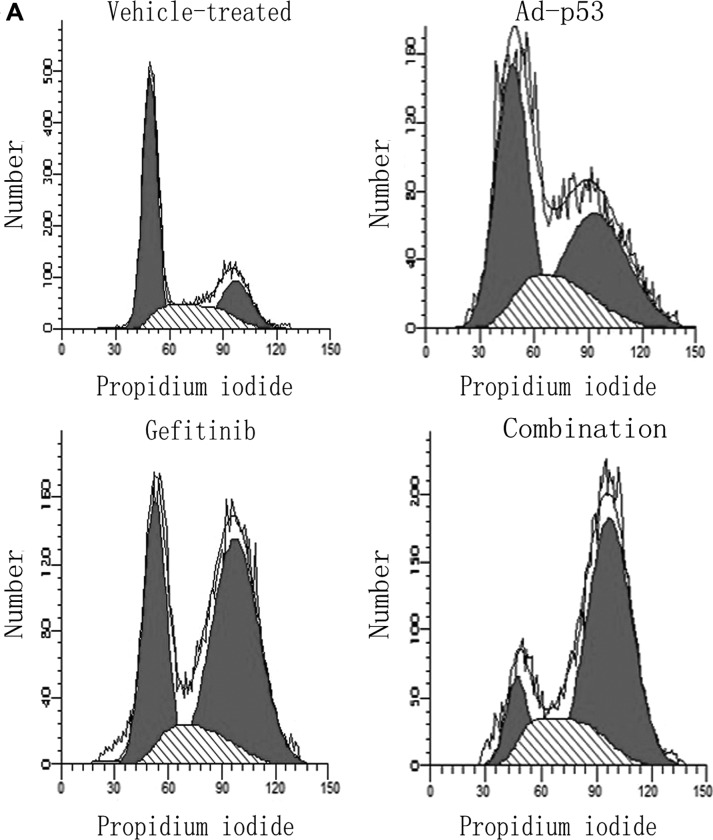

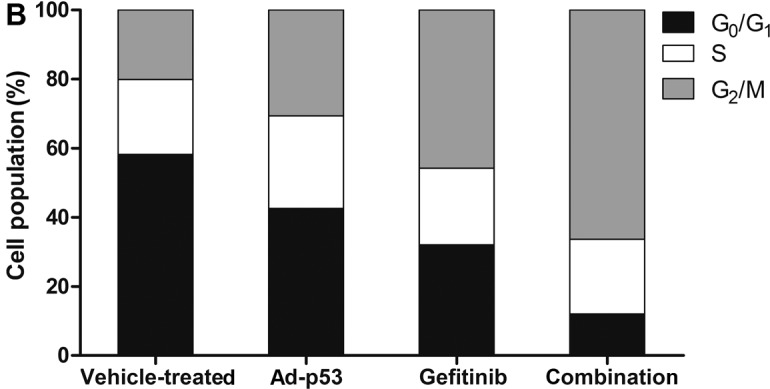
Ad-p53 infection enhances G_2_/M arrest induced by gefitinib. MDA-MB-468 cells were treated with 3 μM of gefitinib alone, MOI of 100 of Ad-p53 alone or in combination. After 48 h, cell cycle distribution was analyzed by flow cytometry at the indicated time. The graph is based on three independent measurements with similar results. Ad-p53, recombinant human p53 adenovirus; MOI, multiplicity of infection.

MDA-MB-468 cells were treated with either drug alone or in combination with Ad-p53 for 48 h. According to flow cytometry, the apoptosis rate in the Ad-p53, gefitinib, combination and vehicle-treated group was 17.4, 20.5, 32.6 and 8.5%, respectively. Treatment with Ad-p53 or gefitinib alone slightly induced cellular apoptosis (Fig. 3). The combined treatment increased cellular apoptosis to 4-fold when compared with the vehicle-treated control (Fig. 3). As shown in Fig. 4, gefitinib induced G_2_/M phase arrest from 21.9 to 45.4% compared to the vehicle-treated groups; G_2_/M phase increased to no more than 31.5% after exposure to Ad-p53. In comparison, when the combined treatment was performed, G_2_/M arrest was enhanced evidently from 21.9 to 65.3%.

### Effect of treatment with Ad-p53 and/or gefitinib on intracellular signaling

**Figure 5:**
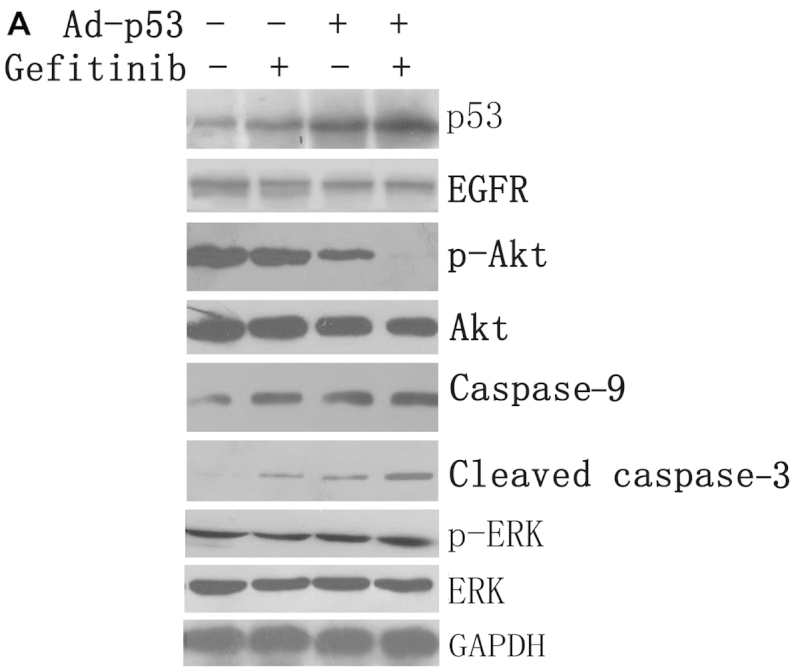

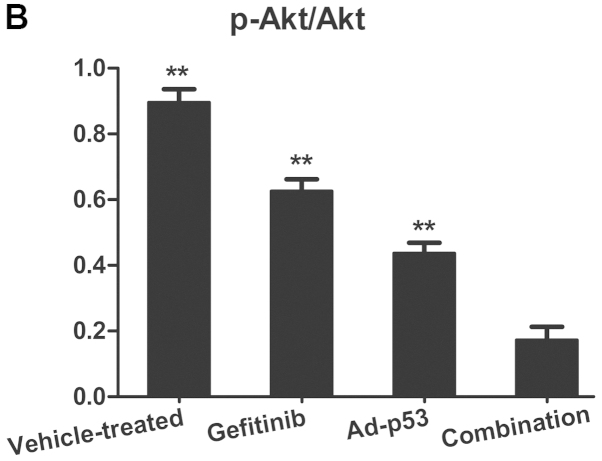

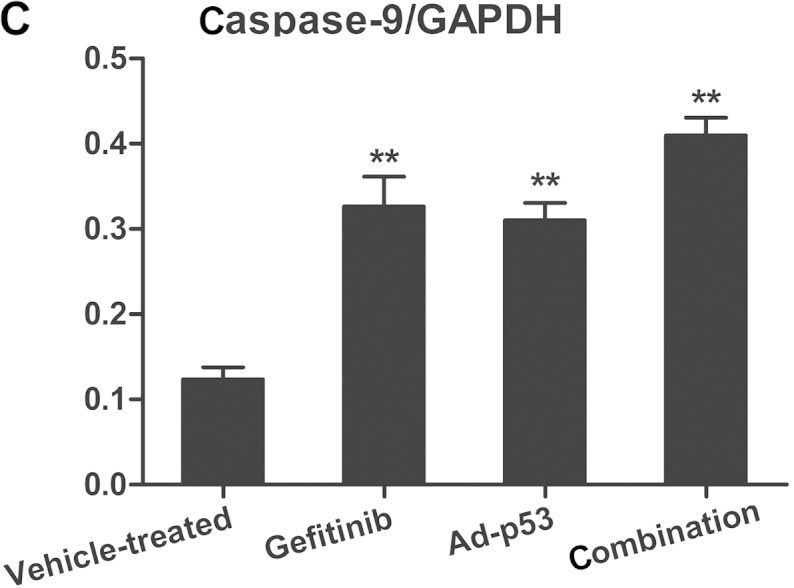

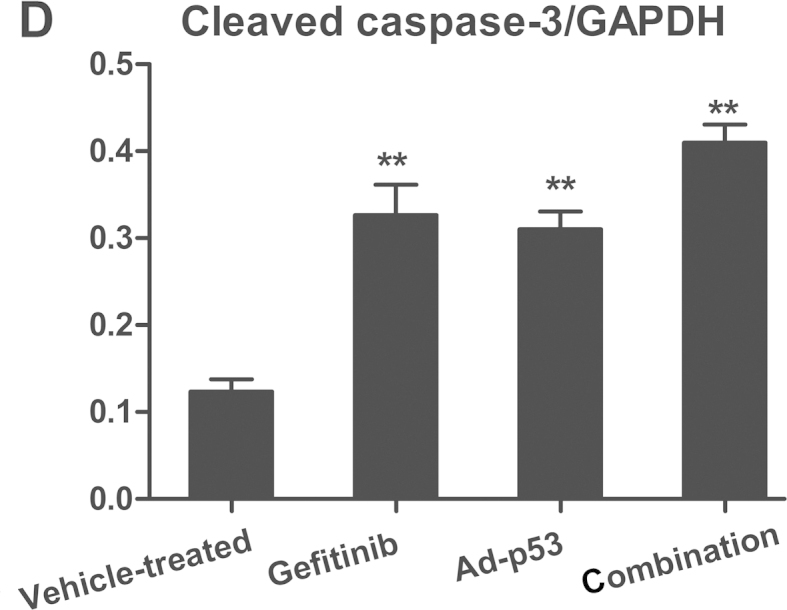
Combination of Ad-p53 and gefitinib suppresses the Akt pathway in MDA-MB-468 cells and increases the activity of caspase cascade protein. Cells were treated with Ad-p53 (MOI of 100), gefitinib (3 μM), alone or a combination for 48 h. Cell lysates were analyzed via western blotting using the indicated antibodies. GAPDH was used as a loading control. (A) p53 and EGFR expression was detected in the MDA-MB-468 cells by western blotting. Ad-p53 and gefitinib in combination significantly downregulated p-Akt and upregulated caspase-9 and cleaved caspase-3. ERK and p-ERK showed little change among the four groups. (B) Relative expression of p-Akt, (C) caspase-9 and (D) cleaved caspase-3 was evaluated by ANOVA. Tukey’s multiple comparison was applied to compare two subsequent samples. Data represent means ± SEM from three independent experiments. ^**^P<0.01. Ad-p53, recombinant human p53 adenovirus; MOI, multiplicity of infection; EGFR, epidermal growth factor receptor; p-Akt, phosphatidylinositol-3 kinase; ERK, extracellular signal-regulated kinase; p-ERK, phosphorylation of ERK.

Ad-p53 or gefitinib, as a single agent, produced a slight reduction in the levels of p-Akt (S473) in the MDA-MB-468 cells (Fig. 5). Surprisingly, combined treatment synergistically produced a significant reduction in p-Akt (S473) (Fig. 5). The expression levels of caspase-9 and cleaved caspase-3 were low in the vehicle-treated cells. In contrast, when Ad-p53 or gefitinib was used as a single agent, the levels of caspase-9 and cleaved caspase-3 increased (Fig. 5). Nevertheless, when they were administered in combination, the levels were markedly increased. However, none of the treatments led to an obvious change in ERK and p-ERK. These results suggest that Ad-p53 may enhance the sensitivity of MDA-MB-468 cells to gefitinib by blocking the PI3K/Akt pathway rather than the Raf/MEK/ERK pathway.

### Efficacy of the treatments on the MDA-MB-468 xenografts in vivo

**Figure 6:**
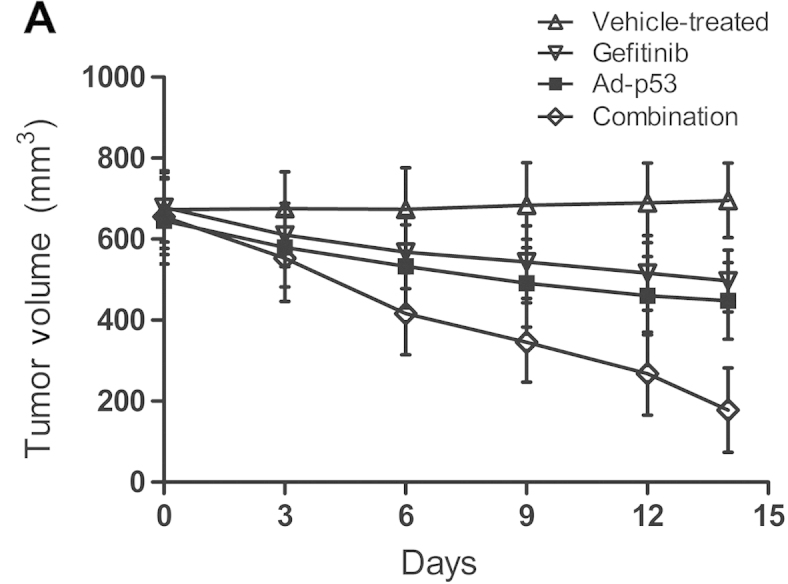

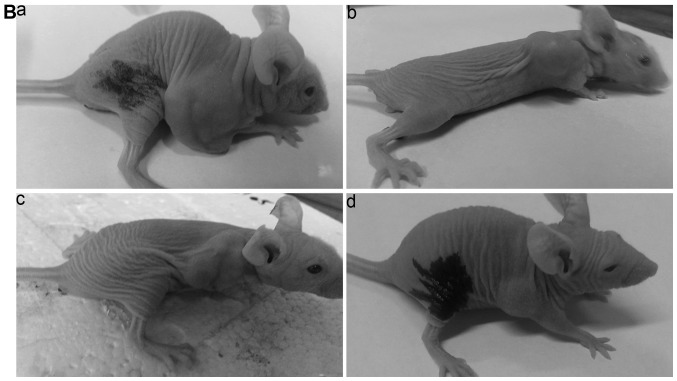
Ad-p53 and gefitinib combination therapy significantly reduces tumor volume. The analysis of the xenograft tumor volumes was performed according to Materials and methods. (A) Tumor inhibition rate was calculated after MDA-MB-468 cells were treated with Ad-p53 and/or gefitinib. Data represent means ± SEM from three independent experiments. (B) A slight decrease in size was observed when the nude mice were treated with either (b) Ad-p53 or (c) gefitinib. (d) Tumor volume was significantly decreased after Ad-p53 and gefitinib were administered in combination, and (a) the volume of the vehicle-treated xenografts increased slightly. Ad-p53, recombinant human p53 adenovirus.

Ad-p53 and gefitinib were administered to the MDA-MB-468 xenografts and tumor volume was measured periodically. Ad-p53 or gefitinib alone caused slight tumor volume shrinkage (Fig. 6). The tumor inhibition rate (TIR) in the Ad-p53, gefitinib and combination group was 35.7, 28.7 and 74.4%, respectively. The xenografts were significantly reduced in size when Ad-p53 and gefitinib were administered simultaneously (Fig. 6).

## Discussion

p53 is a tumor suppressor gene known to be the most commonly altered gene in human cancer (21). Due to the aggressiveness of cancer cells with p53 mutation, many studies have been devoted to eradicating the mutant p53 through a variety of methods such as isolating the interaction between p53 and p63 with peptide aptamers (PA) (22), restoring the function of p53 (23), and disrupting the interaction between EGFR and colony stimulating factor 1 receptor (CSF-1R) (24). All these approaches may remedy the defect of p53 dysfunction. The p53 mutation is kept at a low level in breast cancer, yet mutant p53 is more prevalent in TNBC (25). Perhaps this is the reason why TNBCs progress rapidly and have a poor prognosis. MDA-MB-468 cells harboring EGFR overexpression, as a gefitinib-resistant model, have been demonstrated in different studies (26,27).

Small-molecule tyrosine kinase inhibitors (TKIs) against EGFR have been evaluated in breast cancer. Baselga *et al* (9) reported that gefitinib demonstrated minimal single-agent activity when treating metastatic breast cancer. Resistance to EGFR inhibitors present a huge obstacle to breast cancer patients (3). In the present study, in order for us to explore whether p53 increases the sensitivity of an EGFR inhibitor, Ad-p53 and EGFR TKI gefitinib were used to treat a TNBC cell line. Notably, the sensitivity of MDA-MB-468 cells to gefitinib was significantly increased when they were pretreated with Ad-p53. The cell proliferation assay indicated that when cells were treated with gefitinib, the IC_50_ value of Ad-p53-infected cells was almost half as much as the vehicle-treated cells (Fig. 1, Table I). Furthermore, the results of the *in vitro* experiments, such as the clonogenic and apoptosis assays and cell cycle distribution, revealed that p53 enhanced the sensitivity to gefitinib by inhibiting colony formation (Fig. 2), by regulating cellular apoptosis (Fig. 3) and by inducing cell cycle arrest (Fig. 4).

MDA-MB-468 cells pretreated with Ad-p53 showed enhanced sensitivity to gefitinib with downregulation of p-Akt, according to western blotting results, while ERK and p-ERK exhibited little or no change. Both the PI3K/Akt and Raf/MEK/ERK pathways are downstream of EGFR activation. The former can resist apoptosis, while the latter is involved mostly in anti-apoptosis as well as cell proliferation (28,29). MDA-MB-468 cells possess an elevated level of p-Akt, and their persistent activation of p-Akt is relevant to their resistance to EGFR inhibitors (12). Previous studies suggest that p53 may participate in the modulation of the PI3K/Akt and Raf/MEK/ERK pathways in cancer cells (30–32). Our data, however, indicate that Ad-p53 may interfere with the PI3K/Akt pathway rather than the Raf/MEK/ERK pathway, leading to an increase in the sensitivity to gefitinib. Moreover, caspase-9 is a crucial component of the apoptosis pathway, and activated caspase-9 initiates the caspase cascade by driving the activity of downstream caspases such as caspase-3, -6 and -7. In the present study, caspase-9 and cleaved caspase-3 increased synergistically when MDA-MB-468 cells were exposed to both Ad-p53 and gefitinib in comparison to the single agent treatment, suggesting that caspase-mediated apoptosis was triggered in this TNBC treatment. Similarly, Chang *et al* (33) reported that gefitinib induced apoptosis via a p53-dependent pathway in a lung cancer cell model, which was accompanied by the upregulation of pro-apoptotic molecules (such as Fas and PUMA) and the downregulation of anti-apoptotic molecules (such as XIAP and survivin). Therefore, the synergistic effect of the combined treatment could be attributed to the effect of gefitinib in triggering caspase-dependent apoptosis via inhibiting the PI3k/Akt pathway potentiated by Ad-p53. Recently, Yu *et al* (34) reported that caspase-dependent apoptosis and inactivation of the PI3K/Akt pathway were the main apoptotic mechanisms of human gastric carcinoma AGS cells. Further studies of expanded TNBC cells should be conducted in order to obtain more detailed mechanisms related to the dysfunction of the PI3K/Akt pathway and caspase cascade activation.

Ad-p53 is effective for treating numerous malignancies, including colon, glioma, lung, ovarian and head and neck tumors (35–39). In the present study, the *in vivo* experiment was designed to mimic a clinical situation in order to document whether Ad-p53 and gefitinib together synergistically inhibit the growth of transplanted breast tumors in nude mice. According to our results, the combination of Ad-p53 and gefitinib significantly alleviated the bulk of the tumor burden in the nude mice. These results may pave the way for the clinical treatment of patients who are resistant to EGFR TKIs. Moreover, when these two agents are used together, they not only compensate for the shortcomings of one another, but also maximize their benefit. For example, their effective combination could potentially reduce EGFR inhibitor-related side-effects, while exhibiting better antitumor abilities, thus enhancing the quality of life of these patients. To further judge the clinical applicability of both agents, clinical trials should be conducted in follow-up studies.

In conclusion, finding an effective therapeutic regimen to cure TNBCs has become an urgent need; TNBCs are diagnosed in nearly 20% of breast cancer patients, most of whom are young and have a high rate of recurrence. In the present study, we demonstrated the feasibility of combining Ad-p53 and the EGFR inhibitor gefitinib to treat TNBC cells, which are relatively resistant to gefitinib. Wild-type p53 has a good application perspective for sensitizing EGFR inhibitors both *in vitro* and *in vivo*. This may stimulate researchers to restore wild-type p53 in order to enhance the effectiveness of EGFR targeted therapies.

## Figures and Tables

**Table I:** IC_50_ value of MDA-MB-468 cells for gefitinib with or without Ad-p53.

Group	IC_50_ gefitinib (μM)
Ad-p53	4.3±0.4^a^
Vehicle-treated	8.5±0.3

a P<0.05, statistical significance by Student’s t-test.

Ad-p53, recombinant human p53 adenovirus.
